# Medical Items

**Published:** 1895-05

**Authors:** 


### *MEDICAL ITEMS.*

Mr. Tait says the medical profession is badly afflicted with *bacilliphobia.* The worst victim that we have heard of is Dr. F. P. Mann. He advises the constant wearing of a "thin pledget of cotton" in the nose to keep out bacteria.

The National Association of Military Surgeons meets in Buffalo, N. Y., May 21 to 23d. The President is Dr. George N. Sternberg, Surgeon-General U. S. Army; the Secretary, Dr. Eustathius Chancellor, 505 Olive Street, St. Louis.

The Medico-Legal Society of New York announces that it will hold a Medico-Legal Congress at or near the City of New York during the last week of August or first week of September, 1895 (time and place to be hereafter announced), open to all students of medical jurisprudence. A formal and official circular will be issued later. H. W. Mitchell, M.D., President; Clark Bell, Esq., Secretary.

It will be remembered that several months ago the notorious quack concern, the Amick Consumption Cure Company, of Cincinnati, brought suit against Dr. J. E. Reeves, of Chattanooga, for exposing their business. The suit has just been finished, the jury rendering a verdict in favor of Dr. Reeves, with the costs fixed upon the Amick Company. We are satisfied that this is entirely as it should be. Served 'em right.

Life insurance companies depend for their success upon the skill, intelligence, and character of the medical men who examine for them. It behooves them to treat the medical profession in a generous manner. Yet the New York Life has just reduced its fee for examination to three dollars, and the Equitable, and we presume other companies, send out circulars begging for free information regarding the character of those whom they employ.—*Medical Record.*

---

THE Buffalo (N. Y.) *Medical and Surgical Journal* is about to celebrate its semi-centennial. It was established fifty years ago by the late Austin Flint when there were only about a dozen journals in the country. Its career has been one of continuous prosperity and success. Under the able management of Dr. William Warren Potter it begins its second fifty years increased in size. We offer our best wishes for another half century of prosperity.

---

ACCORDING to our inflammatory contemporary, the *Texas Medical Journal,* a sure cure has been discovered for ophthalmia neonatorum by a Texas legislator. When the bill to prevent blindness, by compelling early attention to sore eyes in the new born, was before the legislature, an old fellow, calling himself "doctor," hooted at the idea, and said "a little mother's milk dropped in the baby's eye would cure it sure pop, every time." It killed the bill.

---

"THE meanest man I know of lives in Kansas," said a St. Louis physician. "He is a farmer, worth a cool hundred thousand. His wife was taken suddenly ill, and he came to town to consult me about her case. I told him that I could not prescribe intelligently without seeing the patient, but he declined to incur the expense of a visit. I charged him $1 for the prescription, and he spent half an hour trying to beat me down to 90 cents. He made me write the prescription in English, then bought the drugs and compounded it himself to save the apothecary's fee. One of the ingredients was capsicum. He thought he had some at home, but was mistaken, and had to come back to town, a distance of four miles, for it. By the time he had succeeded in saving about twenty cents, and wasting two dollars' worth of time, his wife was dead and the medicine a loss on his hands. That so wore on him that he fell ill. He took the medicine prepared for his wife, but that only aggravated his malady. When he finally recovered he sued me for $10,000, and was beaten and had to pay costs. He then went before the grand jury and tried to have me indicted for malpractice."

This man is about on a par with the fellow who takes a medical journal for several years, and when asked to pay for it drops it back in the office and has it marked "refused."

---

OUR friend, Mr. Angus E. Orr, has shown us the following old medical bill and receipt found among some family papers. It illustrates very well the therapeutics of ninety years ago:

MR. JAMES ORR TO ROBERT MOORE, DR.

| | | |
|---|---|---|
| July 16, 1804. | Visit, attendance and venesection in his own case continued fever | $ 3 00 |
| | Emetic of ipecac and tartar emetic | 25 |
| | Best red bark, ℥ iiss | 1 25 |
| | Diaphoretic powders, No. 8 | 25 |
| 21. | Visit and laxative and diaphoretic powders, ℥ i | 75 |
| 23. | Visit and febrifuge powders No. 3 | 1 00 |
| | Drops, diaphoretic and restorative, ℥ i | 50 |
| | Blistering plasters, No. 2 | 1 00 |
| 26. | Diaphoretic drops | 50 |
| | Paregoric and lavender comp., ℥ iss | 75 |
| | Flor. chamomil, ℥ i | 25 |
| Aug. 2. | Diaphoret. drops, ℥ i | 50 |
| | | $10 50 |

Jan. 28, 1805. Received the amount of the above account in full of Mr. James Orr.

ROBERT MOORE.

